# Sacral Anatomical Orientation in the Lebanese Population

**DOI:** 10.1155/2020/4292384

**Published:** 2020-04-07

**Authors:** Joseph Maalouly, Fouad Jabbour, Elias Saidy, Georgio Lati, Gerard El-Hajj, Dany Aouad, Rami Ayoubi, Alexandre Nehme

**Affiliations:** ^1^Department of Orthopedic Surgery and Traumatology, Saint George Hospital University Medical Center, Balamand University, P.O. Box 166378, Achrafieh, Beirut 1100 2807, Lebanon; ^2^Department of Radiology, Saint George Hospital University Medical Center, Balamand University, P.O. Box 166378, Achrafieh, Beirut 1100 2807, Lebanon

## Abstract

**Introduction:**

PI is currently used as the gold standard measurement in spinopelvic anatomy. There is a need for a reliable method to calculate sacral anatomic orientation (SAO) independent of posture and to establish its association with PI, which was previously established in a single study (Peleg et al., 2007). Therefore, the aim of our study is the application and verification of this association on a Lebanese sample.

**Methods:**

Methods for measuring SAO and PI on living individuals are described. The study was carried out on 200 adult individuals using CT 3D images (volume-rendering method). Reliability (intratester and intertester) was evaluated using the intraclass correlation test. A regression analysis was carried out to evaluate the association between the two measurements.

**Results:**

There were 103 females (51%) and 97 males (49%) with a mean age of 58.68 ± 19.6 years (min = 20; max = 93). The mean SAO and PI in our population were found to be 52.65° (SD = 8.16°) and 59.08° (SD = 12.53°), respectively. SAO and PI measurements were highly correlated (Pearson correlation test; *r* = −0.296, *P* < 0.0001 for our general population). PI can be predicted via SAO, i.e., SAO = (−0.193 × PI) + 64.057.

**Conclusions:**

SAO may be an important tool, alongside PI, in defining the sagittal shape of the spine and useful for understanding its association with spinal diseases as they are not affected by postural changes.

## 1. Introduction

A previous study by Peleg et al. [1] has already established an association between SAO and PI. But, in order to correctly assess SAO or PI on individuals of different ancestry or from different populations, it is fundamental that the method be tested on the specific population one is working on. Therefore, the aim of the present study is the application and verification of the already described method of measuring SAO and its association with PI on a Lebanese population sample.

Pelvic orientation (PO) is considered a key factor in spinal shape and has been shown to significantly influence spino-pelvic balance in normal and pathologic conditions. This finding has important clinical and anthropologic implications and has fostered a renewed interest in the radiologic evaluation of PO. Usual parameters of its evaluation are sacral slope (SS), pelvic tilt (PT), and pelvic incidence (PI) [2, 3].

SS is defined as the angle created between a line running parallel to the superior surface of the first sacral vertebra and a horizontal line. PT is defined as the angle between the vertical line and the line joining the middle of the sacral endplate and the axis of the femoral heads. PI is defined as the angle between the line perpendicular to the sacral endplate at its midpoint and the line connecting this point to the joined axis of the femoral heads [4]: PI = SS + PT.

PI is the gold standard measurement, and it has been proven many times to correlate with spinal parameters like lumbar lordosis and thoracic kyphosis.

Increased PI and decreased pelvic lordosis were found in low-grade isthmic spondylolisthesis [4, 5]. In the past decade, extensive research was conducted on this subject, and PI and pelvic lordosis were found correlated with the severity of spondylolisthesis [6]. Recently, Mays [7] based on observation and measurements taken on the skeletal material concluded that high PI may have heightened the risk of developing pars interarticularis defects.

The advantage of PI over SS and PT is that its angular values are unaffected by posture. Its disadvantage is that individuals with similar SS may manifest different values of PI due to a different location of the sacrum within the pelvis and/or dissimilarity in the anteroposterior size of the superior surface of the first sacral vertebra. Moreover, as angles in both methods are measured from standing lateral radiographs, the angle obtained may be affected by the superimpositioning of anatomic structures, magnification of structures further from the film. Recently, CT scan with 3D reconstruction has become more widely available and is helpful in determining spinal pathology.

Those disadvantages and the clinical significance of those angles were behind the search for a new method to calculate sacral anatomic orientation (SAO) independent of posture and to establish the association between PI and SAO.

## 2. Materials and Methods

The study sample consisted of 200 randomly selected pelvic CTscans performed in the radiology department of Saint George Hospital, Beirut, Lebanon, between January 2006 and December 2007. All selected individuals were adults (>20 years) from Lebanese origin (verified by phone). Information on age and sex was recorded for every individual when the CT scan was done. Informed written consent was obtained from all patients. There were 103 females (51%) and 97 males (49%) with a mean age of 58.68 (SD = 19.6) years (min = 20; max = 93).

After acquisition of the data on a General Electric CT scan machine, with a thickness of cut of 1 to 3 mm, each set of data was copied on a CD for each individual and subsequently imported on a Sony Vaio laptop computer. Each examination was then opened using the Amira 4.1 volume rendering software. Every stack of images was imported through Amira and opened first as three orthogonal slices in the 3 spatial planes. This allows using the cropping module which narrows the region of interest to the pelvis excluding thus the lumbar spine and any additional artifact. An isosurface was then generated using the Amira 3D rendering module which allows obtaining a volumetric representation of the pelvis. 2D and 3D measurements of distances and angles were then carried on the buildup. All measurements were verified by three observers.

### 2.1. Measurement of SAO Angle

**Figure 1:**
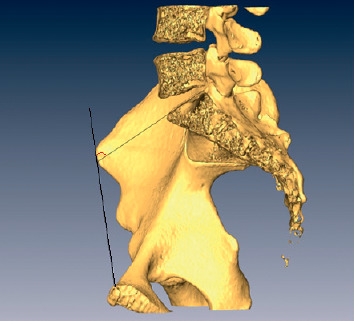
Sacral anatomic orientation.

SAO is the angle created between the intersection of a line running parallel to the superior surface of the sacrum and a line running between the anterior superior iliac spine (ASIS) and the anterior-superior edge of the symphysis pubis (Figure 1, angle *α*). It was measured using Amira 4.1 using the following steps. Using the module isolines, a sagittal plane was created at the middle of the sacrum and perpendicular to the sacral plate. The pelvis was rotated until we obtained complete superimpositionning of both hemipelvises verified with the perfect alignment of both iliac crests and both anterior inferior iliac spines. The hemipelvis at the front was then cropped at the mid-sagittal isoline plane. This allowed exposure of the sacral plate and the drawing of a line running parallel to the superior surface of the sacrum, as well as another line running between the anterior superior iliac spine (ASIS) and the anterior-superior edge of the symphysis pubis. The 2D angle formed between those 2 lines was measured and recorded for each individual.

### 2.2. Measurement of PI Angle

**Figure 2:**
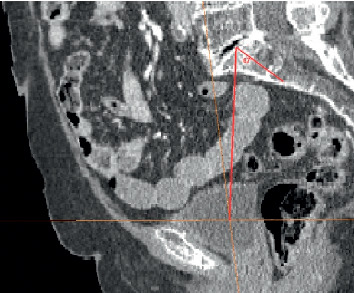
Pelvic incidence.

PI is defined as the angle between the line perpendicular to the sacral plate at its midpoint and the line connecting this point to the middle of the axis connecting the centers of both femoral heads (Figure 2). To be able to obtain exactly the line connecting the centers of both femoral heads, we used the intersection of two orthogonal planes (coronal and horizontal) cutting simultaneously both femoral heads into identical halves. The pelvis was then rotated until we obtained complete superimpositionning of both hemi pelvises, which allowed the projection of the line connecting the centers of both femoral heads as a single point. A line was then drawn on the sagittal plane to connect this point to the center of the sacral plate and also the perpendicular to the sacral plate at its midpoint. The 2D angle formed between those 2 lines was measured and recorded for each individual.

### 2.3. Statistical Analysis

In order to determine the association between SAO and PI, Pearson correlation analysis was done and then a linear regression analysis was carried out. The measurements were taken by one observer (AN – *n* = 200). The intraclass correlation coefficient was used to determine the intratester reliability of the measurements. An independent *t*-test was done between the spondylolisthesis group (grades 1 and 2) and the control group.

## 3. Results

### 3.1. SAO and PI Reliability

 
*SAO.* Reliability was high. Intraclass correlation coefficient for intratester reliability was 0.967 (*P* < 0.001). Intertester reliability was 0.89 (*P* < 0.001).
 
*PI*. Reliability was similarly high. Intraclass correlation coefficient for intratester reliability was 0.971 (*P* < 0.001). Intertester reliability was 0.82 (*P* < 0.001).

### 3.2. Assosciation between SAO and PI

**Figure 3:**
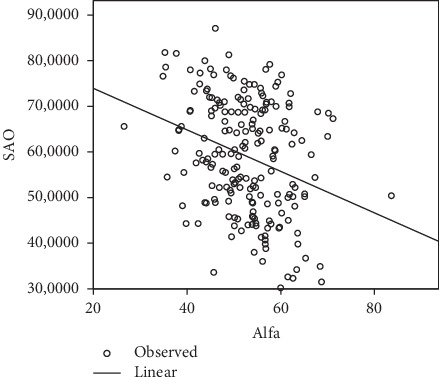
Association between PI (alfa) and SAO.

SAO and PI were correlated (Figure 3) negatively with a pearson correlation coefficient *r* = −0.296 (*P* < 0.0001). PI and SAO can be deduced from each other according to the following formulas: SAO = (−0.193 × PI) + 64.057; PI = (−0.455 × SAO) + 83.05.

### 3.3. Comparison of SAO and PI Values between Spondylolisthesis Population and Control Group

The mean value of SAO is significantly lower in the spondylolisthesis group while the mean of PI is higher in the spondylolisthesis group (Table 1). The Student *t*-test, after assuming equal variances according to Levene's test, shows significant difference between the two groups for SAO (Table 2).

## 4. Discussion

The present study validates the described method for assessing SAO, based on the anatomic position of the human pelvis. SS and PT vary with posture and they are measured relative to a horizontal line and a vertical line, while SAO is measured relative to the ASIS-pubis line located in humans on the same coronal plane. Thus, it can be measured constantly despite postural change.

The finding that SAO is correlated with PI lends validity to the former as a measurement that takes into consideration not only the physiologic aspect of sacral orientation (measured relative to the axis of gravity), but also the anatomic ones. Today, PI is the main parameter measured for pelvic morphology and its association with spinopelvic alignment and spinal deformities [4]. Nonetheless, our findings show a negative correlation, an inverse linear curve, between SAO and PI in a significant part of the Lebanese adult population, and PI cannot be explained by SAO alone. To our knowledge, no such study was done using pelvis CT scans for comparison of SAO and PI and their correlation in a living Lebanese population.

Berthonnaud et al. [3], in a review of 160 asymptomatic adult volunteers, have shown that the pelvis and spine in the sagittal plane can be considered as a linear chain linking the head to the pelvis where the shape and orientation of each anatomical section is tightly connected and affects the neighboring section to preserve a stable posture with the least amount of energy expenditure. Changes in shape or orientation at one level will directly affect the adjacent segment. Knowledge of these normal relationships is of prime importance for the comprehension of the sagittal balance in normal and pathologic conditions of the spine and pelvis. The pelvic shape, which was quantified by the PI angle and now by the SAO angle, determines the position of the sacral end. The spine reacts to this position by changing the lumbar lordosis (LL) accordingly; the amount of lordosis increases as the SS increases to maintain the balance of the trunk in the upright position.

Thus, the decision as to which angle is more appropriate depends largely on the question asked. For example, issues relating to spinal malalignment could benefit from both parameters.

We speculate that in the etiology of spondylolysis and spondylolisthesis, pelvic incidence is a significant variable. By definition, a vertically oriented sacral endplate is associated with a high grade of pelvic incidence [8]. The vertical sacral endplate increases the shear forces acting across L5-S1 disc, which will cause increased traction on the L5 pars interarticularis. This, in turn, may break the pars under these conditions due to the traction effect. Therefore, slipping of the vertebra anteriorly will ensue. It has been demonstrated that increased slip angle, female sex, a vertical orientation of the L5-S1 intervertebral disc, and early age at the onset of symptoms are associated with progression of spondylolisthesis [9–11]. In our study, SAO decreases in the spondylisthesis group as opposed to the PI which increases (Table 1). This further demonstrates the inverse relationship between the two parameters. In addition, a significant difference of SAO values between both groups was noticed by using the Student *t*-test (Table 2).

In contrast, there are less shear forces acting on the pars when the L5-S1 junction is oriented more horizontally, such as when the sacral slope is less than approximately 40° and lower pelvic incidence [8].

Although modifications in human posture do not affect the angular values of PI and SAO, the most significant distinction between the two measurements is that PI portrays pelvic morphology rather than pelvic orientation, while SAO defines both. Furthermore, SAO angle has an inverse linear relationship with PI [12].

Peleg et al.'s hypothesis is that the curves of the spine must adjust to the orientation of the sacrum's body [13]. In reality, this means that a horizontal sacrum results in an almost vertical orientation of the superior disk surface of S1. To cope with such an orientation of the disk surface; if a vertical spine is to be maintained, the lumbar lordosis must increase. Therefore, the more horizontally oriented the sacrum, the deeper the lordosis [13]. This, in turn, results in an increase in the lever arm of the lumbar extensors.

Several articles reported in the literature support the above hypothesis. For instance, it has been shown that the final segmental spinal alignment starts with increased lumbar lordosis in adolescence especially during growth spurt ages 13 to 15 accompanied by a more horizontally oriented sacrum [14]. Roussouly and Pinheiro-Franco [15] demonstrated that spinal curve shapes in adults are associated with PI grade. For example, an increase in lordosis is associated with a high PI.

The importance of these findings lies in emphasizing the role of sacral orientation in establishing normal spinal configuration. Once epiphyseal rings ossification is complete for the vertebral bodies (at 14–16 years of age), no more changes can occur [16]. Adolescent growth spurt studies show that anterior vertebral development exceeds posterior vertebral growth, leading to reduced thoracic kyphosis and increased lumbar lordosis [14, 17, 18].

As we now know, the benefit of using PI and SAO is that their values are unaffected by posture. The main drawback from using PI is that certain individuals with similar SS values may have different values of PI due to a variable location of the sacrum within the pelvis with the possible addition of dissimilarity in the anteroposterior size of the superior surface of the first sacral vertebra. Furthermore, as angles in both methods are usually measured from standing lateral radiographs, the angle obtained can be affected by the overlap of anatomic structures, magnification of structures further from the film. In our series, the measurements of PI and SAO were made on CT scan with 3D reconstruction which benefits from the precision associated with the imaging modality chosen [19–21].

## 5. Conclusion

To sum up, we think that there is a connection between spinal alignment and sacral orientation as demonstrated by the inverse linear curve, similar to Peleg et al. This demonstrates that SAO changes with PI and it can be used across different populations. According to our data and the literature review, it is the orientation of the sacrum that helps dictate the shape of the spine. Therefore, a combination of both PI and SAO, as both parameters are not affected by postural changes, help in gaining a thorough understanding of the spino-pelvic pathologies, including spondylolisthesis. Further studies are warranted for proving the correlation between SAO, lumbar lordosis, and thoracic kyphosis, which has been proven for PI.

## Figures and Tables

**Table 1:** Group statistics of SAO and PI between control and spondylolisthesis groups.

Group statistics	Spondylolisthesis	*N*	Mean	Std. deviation	Std. error mean
SAO	Yes	20	44.8944	8.71987	2.17997
	No	180	53.3404	7.72541	.57582
PI	Yes	20	62.9688	11.60263	2.90066
	No	180	58.7667	12.42240	.92591

**Table 2:** Student *t*-test and Levene's test for SAO and PI between control and spondylolisthesis groups.

	Levene's test for equality of variances						
	*F*	Sig.	*T*	*df*	Sig. (2-tailed)	Mean difference	Std. error difference
SAO							
Equal variances assumed	0.516	0.473	−4.147	198	0.000	−8.446	2.03660
Equal variances not assumed			−3.746	17.159	0.002	−8.4465	2.25473
PI							
Equal variances assumed	0.125	0.724	1.303	198	0.194	4.202	3.22466
Equal variances not assumed			1.380	18.197	0.184	4.202	3.04485

## Data Availability

The SAO, PI, and age data used to support the findings of this study are available from the corresponding author upon request.

## Abbreviations

PO: Pelvic orientation
SAO: Sacral anatomical orientation
PI: Pelvic incidence
PT: Pelvic tilt
SS: Sacral slope
CT: Computed tomography
ASIS: Anterior superior iliac spine
LL: Lumbar lordosis.

## Additional Points

*Limitaions*. This is a retrospective study, using only pelvis CT scan of 200 patients for assessment of spinopelvic parameters.
